# Use of a suspended and straightened knee joint position when fixing steel plates can prevent the increase in postoperative posterior tibial slope after open‑wedge high tibial osteotomy

**DOI:** 10.1186/s13018-021-02834-3

**Published:** 2021-11-18

**Authors:** Wenru Ma, Zengshuai Han, Shengnan Sun, Jinli Chen, Yi Zhang, Tengbo Yu

**Affiliations:** 1 Department of Sports Medicine, the Affiliated Hospital of Qingdao University, 59 Haier Road, Laoshan District, Qingdao, 266000 Shandong Province China; 2 Department of Clinical Medicine, Qingdao University, Qingdao, Shandong Province China; 3 Department of Orthopedics, Affiliated Hospital of Qingdao University, Qingdao, Shandong Province China

**Keywords:** Knee osteoarthritis, Open-wedge high tibial osteotomy, Posterior tibial slope angle, Suspended and straightened knee joint position

## Abstract

**Background:**

Posterior tibial slope (PTS) increases after medial open-wedge high tibial osteotomy (OWHTO) is challenging for patients. This study aims to determine whether use of a suspended and straightened knee joint position during the fixing of steel plates can prevent an increase in the PTS after OWHTO.

**Methods:**

This study retrospectively analyzed 112 subjects (122 knees) [34 males, 78 females; mean age 59.1 ± 6.6 (range 48–76) years; mean body mass index 28.06 ± 3.61 kg/m^2^] who underwent OWHTO. A total of 78 knees that were suspended and extended by placing a sterile cloth ball under the ipsilateral ankle during the fixing of steel plates comprised the suspended and straightened knee joint position (SSP) group, and 44 knees that were kept naturally straightened without placing a sterile cloth ball under the ipsilateral ankle during the fixing of steel plates comprised the naturally straightened knee joint position (NSP) group. Patients were clinically assessed according to the visual analog pain scale (VAS), the Western Ontario and McMaster Universities (WOMAC) osteoarthritis index, the Knee Society Scores (KSS) knee and function scores, the Hospital for Special Surgery (HSS) knee scores and the Lysholm knee scores. Radiological assessment was performed according to the changes in the PTS between preoperation, 1-day postoperation, and the final follow-up periods. Ultimately, the difference in postoperative PTS changes between the two groups was statistically analyzed. The median follow-up period was 2.2 years (range 1.6–3.7 years).

**Results:**

In the final follow-up period, significant improvements were observed in the clinical VAS scores, WOMAC scores, KSS knee and function scores, HSS scores and the Lysholm knee scores in both groups (*P* < 0.001), and no difference was found between the two groups. Radiological assessment showed that there was no statistical difference in the preoperative PTS between the two groups. The 1-day postoperative PTS and the most recent follow-up PTS were significantly greater than the preoperative PTS in the NSP group (*t* = − 3.213, − 6.406, all *P* < 0.001), but no significant increase was seen in the SSP group (*P* > 0.05). The increase in PTS in the NSP group was significantly greater than that in the SSP group at the 1-day postoperative (*t* = 2.243, *P* = 0.030) and final follow-up periods (*t* = 6.501, *P* < 0.001).

**Conclusions:**

For OWHTO, the use of a suspended and straightened knee joint position rather than a naturally straightened knee joint position during the fixing of steel plates could effectively prevent the increase in postoperative PTS.

*Level of Evidence*: Retrospective Study Level III.

## Background

High tibial osteotomy (HTO) is a widely accepted treatment for medial compartment knee osteoarthritis in young and active patients [1–4]. HTO achieves the satisfactory clinical results by accurately correcting the force lines of the lower limbs [5–8]. Good surgical effects can be guaranteed within 6–15 years after HTO, including lateral closing-wedge high tibial osteotomy (CWHTO) and medial opening-wedge high tibial osteotomy (OWHTO) [9–11]. Because CWHTO needs to be accompanied by an additional osteotomy of the fibula with potential risk for peroneal nerve injury, OWHTO has gained popularity due to its favorable clinical outcome. However, OWHTO is also associated with an unintentional increase in PTS, which is difficult to avoid [12–14]. PTS has been widely studied for its causal relationship with tibial translation, knee joint stability and anterior cruciate ligament injuries. The physiological range of PTS is between 6° and 10° in normal people [15]. Rodner et al. observed that increasing the PTS by an average of 5.5° was accompanied by redistribution of the location of intra-articular peak pressure, shifting it posteriorly by 24%. They suggested that inadvertent redistribution of contact pressure into this area may be a cause of pain and clinical failure after OWHTO [16]. Various methods have been used to solve this tricky problem, and achieving a wider posterior opening gap than anterior opening gap has been proposed as a key means to prevent an unintentional increase in PTS [4, 13, 17, 18].

**Fig. 1:**
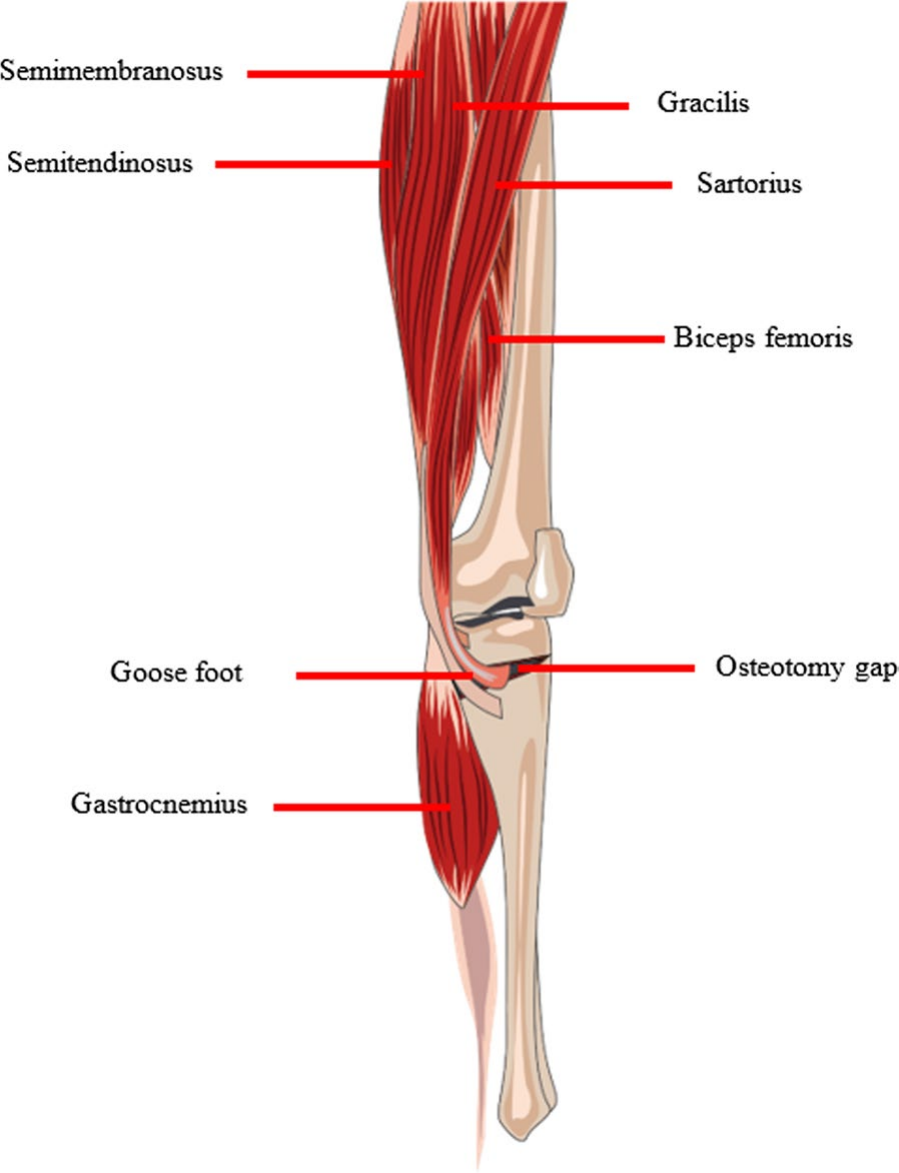
Knee flexors

Clinically, we found that the knee joint position when fixing steel plates is inconsistent during OWHTO. Some surgeons achieve a suspended and straightened knee joint position by raising the heel, while others do not raise the heel to naturally straighten the knee joints, suggesting that OWHTO is not standardized and optimized. The knee flexors, which are strong, may be resistant to posterior cortical gap opening (Fig. 1). Considering that the two knee joint positions may have different effects on the function of knee flexors when fixing steel plates, it is presumed that the use of different knee joint positions may lead to different changes in the postoperative sagittal plane. However, there are no previously published reports on this topic.

The purpose of this study was to investigate the effect of the use of a suspended and straightened or naturally straightened knee joint position on the change in tibial slope in the sagittal plane. Our hypothesis was that, compared with the use of a naturally straightened knee joint position, the use of a suspended and straightened knee position while fixing steel plates is more advantageous in preventing an unintentional increase in PTS after OWHTO.

## Methods

### Study groups

From June 2017 to August 2019, 112 patients (122 knees) were admitted to the Department of Sports Medicine, Affiliated Hospital of Qingdao University and underwent OWHTO. Patients whose knee was kept suspended and straightened during fixing of steel plates comprised the suspended and straightened knee joint position (SSP) group, and patients whose knee was kept naturally straightened comprised the naturally straightened knee joint position (NSP) group.

The inclusion criteria were (1) clinically diagnosed medial compartment knee osteoarthritis with diagnosis of Kellgren-Lawrence grade 3 or 4 knee osteoarthritis at OWHTO period; knee symptoms including severe pain, severely limited activity, and poor quality of life, determined by review of medical records or telephone interviews; (2) healthy knee ligaments and exposure to OWHTO; and (3) a minimum follow-up period of 1 year. The exclusion criteria were (1) fracture of the hinge that occurred after the operation during the follow-up period; (2) loosening or breakage of the steel plate; (3) infection; and (4) loss to follow-up.

Ultimately, 122 knees [from 34 males, 78 females; mean age of 59.1 ± 6.6 (range 48–76) years; mean body mass index (BMI) of 28.06 ± 3.61 kg/m^2^] were enrolled. Of the knees, 78 comprised the SSP group, and 44 comprised the NSP group. Seven subjects were excluded because of hinge fracture or loss to follow-up.

### Osteotomy technique

**Fig. 2:**
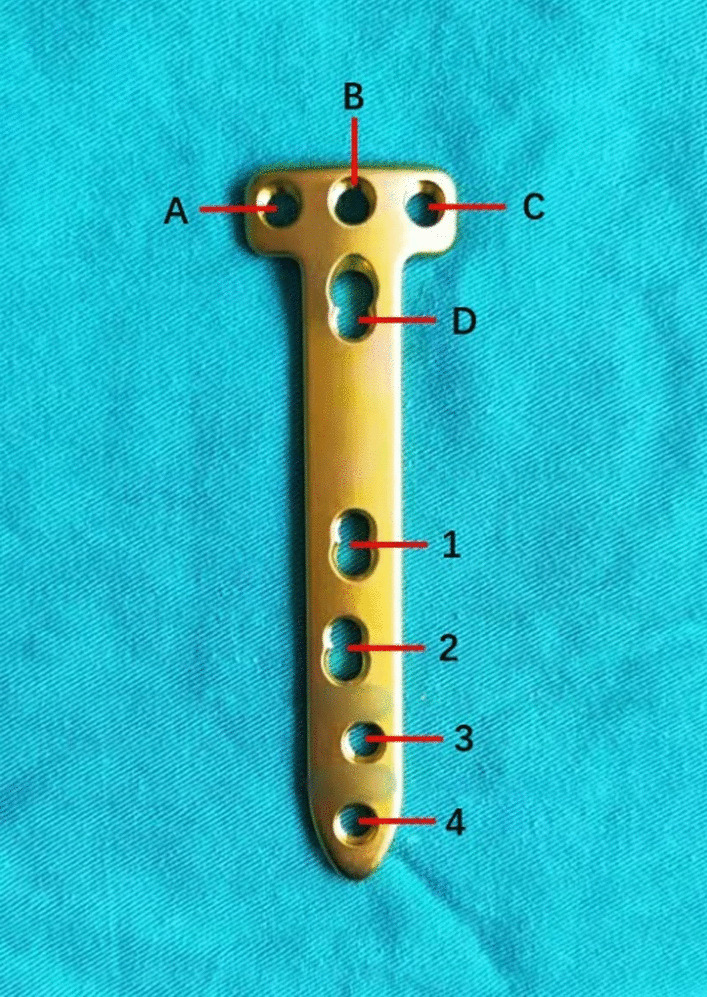
TOMOFIX steel plate

OWHTO was performed by one experienced surgeon. Accurate measurements of varus limb deformities on radiographs were performed for adequate preoperative planning, and a weight-bearing line ratio of 62% on the radiograph was anticipated [16]. Under general anesthesia, a thigh tourniquet was applied while the patient was in the supine position. Open-wedge biplanar osteotomy was performed in all patients using the Lobenhoffer surgical technique [19]. An approximately 5-cm incision was made longitudinally at the 4–5-cm medial portion of the anterior ridge of the tibia. The superficial medial collateral ligament was released subperiosteally from the medial proximal tibia, and the posterior edge of the tibia was fully exposed. A horizontal osteotomy line was designed from the end of the foot toward the fibular head, and an ascending osteotomy line was designed at the medial edge of the tibial tubercle. The target angle between the two osteotomy lines was 110°. TOMOFIX was used for fixation, and other detailed surgical techniques have been described elsewhere [19]. TOMOFIX steel plates were designed to be fixed using eight screws (Fig. 2). First, locking screws *A*, *B*, *C*, *D* were screwed in place. Second, a tension screw was screwed in nail hole 1. This step fixed the position of the steel plates and determined the result of the osteotomy because the fixed part of the steel plate crossed the osteotomy gap. Third, three cortical screws were screwed in place to the other nail holes. Finally, the tension screw in nail hole 1 was changed to a cortical screw. Therefore, the fixing steel plate position mentioned in this study was determined by the second step.

**Fig. 3:**
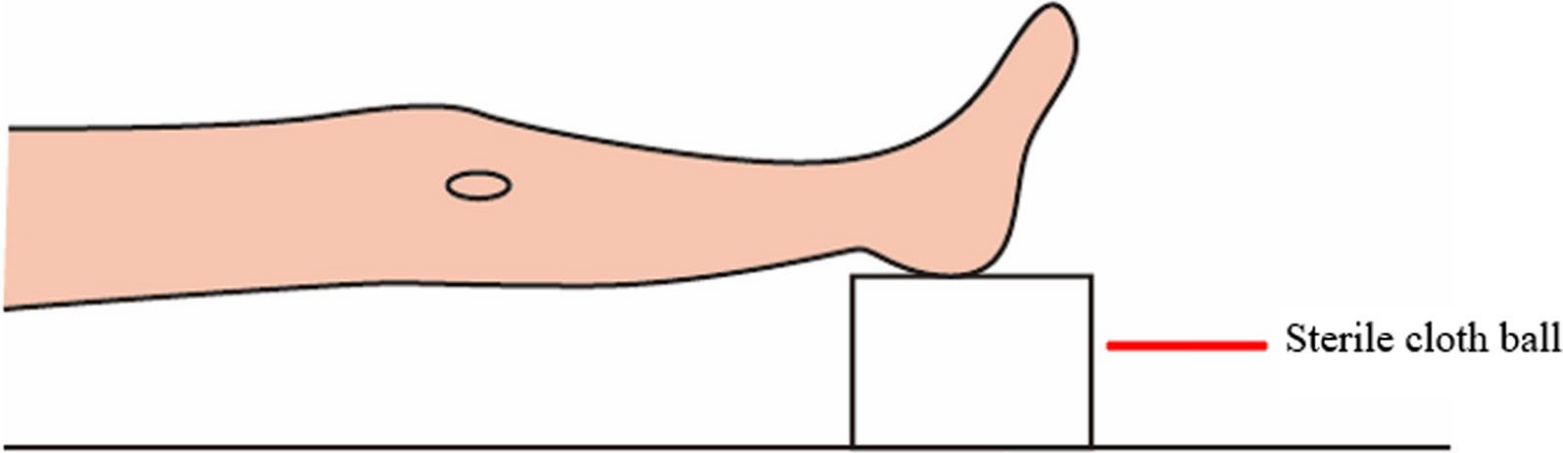
A suspended and extended posture

The knees in the SSP group were suspended and extended by placing a sterile cloth ball under the ipsilateral ankle to raise the ankle when steel plates were fixed (Fig. 3). The knees in the NSP group were in a naturally extended position with the ankle placed directly on the operating table when fixing the steel plates.

### Postoperative treatment and follow-up

In the absence of major contraindications, all patients received treatment for the prevention of deep vein thrombosis and infection in the early postoperative stage, and quadriceps and ankle pump exercises were started to strengthen the isometric contraction of the quadriceps and gastrocnemius. X-ray re-examination was performed at 1 day postoperation. Standard anteroposterior (AP) and lateral radiographs of the knee and weight-bearing full-leg AP radiographs were performed in all patients at the 1-month, 2-month, 3-month, 6-month, 1-year and most recent postoperative follow-up. The lower limbs were allowed to bear weight normally at least 12 weeks after the operation.

### Basic clinical information

The age, sex and body mass index of all patients were recorded. Visual analog pain scale (VAS) at the preoperative stage and the most recent postoperative follow-up were used to evaluate pain symptom relief. The Western Ontario and McMaster Universities (WOMAC) osteoarthritis indexes, the Knee Society Scores (KSS) knee and function scores, the Hospital for Special Surgery (HSS) knee scores and the Lysholm knee scores at the preoperative stage and most recent postoperative follow-up were used to evaluate the improvement of knee joint function.

### Radiographic measurement

The posterior tibial slope (PTS) was the most important index in this study and was measured using lateral radiographs with a digital image viewer. Lateral knee radiographs were obtained for each patients in the preoperative stage, at 1-day postoperation and at the most recent postoperative follow-up.

**Fig. 4:**
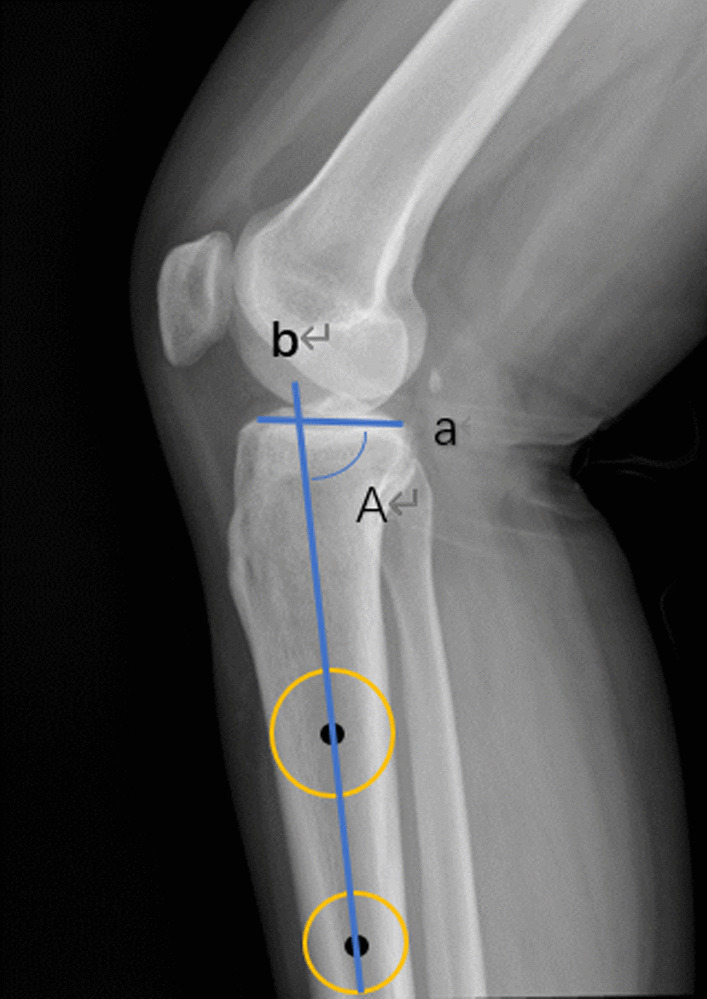
Line b is parallel to the tibial axis and line a is parallel to the tibial plateau. The narrow angle A between line a and b is PTS

PTS was defined as the narrow angle between the proximal tibial anatomic axis and the line tangent to the tibial plateau and was measured according to the method of Omer et al. [20] (Fig. 4). The tibial diaphyseal line was centered through the tibial shaft using digitally generated circles. Two circles with diameters equal to the width of the tibial shaft, which were 15 cm distal to the tibial plateau and 5 cm distal to the tibial tubercle, were digitally drawn on each radiograph. Each circle was sized and positioned so that the anterior and posterior borders of the tibia were tangent to its circumference. A line parallel to the tibial axis was drawn through the centers of these two circles to ensure correct positioning within the center of the tibial shaft (Fig. 4).

Change in 1-day postoperative PTS = 1-day postoperative PTS-preoperative PTS.

Change in most recent follow-up PTS = most recent follow-up PTS-preoperative PTS.

### Statistical analysis

All statistical evaluations were performed using PASW Statistics (25.0, SPSS, Chicago, IL, USA). The Kolmogorov–Smirnov normality test was used before the statistical analyses to determine whether to use a parametric test. Continuous variables conforming to normal distribution are expressed as the mean and standard deviation. Two independent sample t-tests were performed to compare radiological measurements between the SSP and NSP groups, and paired t-tests were performed to analyze the changes in PTS after the operation. Categorical variables are expressed as frequencies (%), and the data from the two groups were compared using the chi-square test. Intra- and interclass correlation coefficients (ICCs) with 95% confidence intervals (CIs) were used to assess intra- and interrater variability. ICC > 0.75 was considered to indicate excellent agreement. A *P* value < 0.05 was considered statistically significant.

## Results

### Basic information

The median follow-up period was 2.2 years, with a range of 1.6–3.7 years. Good to excellent intra- and interobserver variability was achieved for all measurements with an interrater ICC between 0.803 and 0.916 and an intrarater ICC between 0.909 and 0.976.

### Differences in clinical information between the two groups

The t-tests and chi-square tests showed that there were no significant differences in clinical data, i.e., patient age, sex, BMI, and follow-up time, between the two groups (Table 1).

**Table 1:** Comparation of clinical information

	NSP group	SSP group	*t*/*χ*^2^	*P* value
Age	61.9 ± 6.2	58.3 ± 6.6	1.676	0.110
Sex				
Male	12(27.3%)	24(30.8%)	0.05	0.823
Female	32(72.7%)	54(69.2%)		
BMI (kg/m^2^)	27.96 ± 3.75	28.09 ± 3.62	− 0.106	0.916
Preoperative PTS (°)	9.4 ± 1.9	9.3 ± 2.3	0.166	0.869
Follow-up time (year)	3.2(2.1,5)	2.9(1.6,4.7)	0.771	0.137

BMI, body mass index; PTS, posterior tibial slope

### Preoperative and postoperative VAS scores

Two independent sample t-tests and paired t-tests showed that pain was significantly relieved after the operation, as VAS scores were significantly decreased in both groups (*t* = 9.988, *P* < 0.05; *t* = 45.951, *P* < 0.05). There was no significant difference in the preoperative or most recent follow-up VAS score between the two groups (*P* > 0.05) (Table 2).

**Table 2:** Comparation of preoperative and postoperative VAS

	*n*	Preoperative VAS	Postoperative VAS	*t*	*P* value
NSP group	44	8.00 ± 1.10	1.91 ± 1.64	9.988	< 0.001
SSP group	78	7.49 ± 0.79	1.36 ± 0.54	45.951	< 0.001
*t*		1.741	1.096		
*P* value		0.088	0.297		

VAS, visual analogue scale/score

### Preoperative and postoperative knee function

Two independent sample t-tests and paired t-tests showed that the function of the knee joints was markedly improved after the operation, as the WOMAC score significantly decreased in both groups (*t* = 14.110, *P* < 0.05; *t* = 42.530, *P* < 0.05), and the KSS knee score (*t* = − 14.320, *P* < 0.05; *t* = − 27.839, *P* < 0.05), KSS function score (*t* = − 12.183, *P* < 0.05; *t* = − 35.669, *P* < 0.05), HSS score (*t* = − 20.923, *P* < 0.05; *t* = − 47.703, *P* < 0.05) and the Lysholm knee scale (*t* = − 8.532, *P* < 0.05; *t* = − 22.226, *P* < 0.05) were all increased in both groups. There was no significant difference in the preoperative or most recent follow-up WOMAC score, the KSS knee score, KSS function score, HSS score or the Lysholm knee scale between the two groups (*P* > 0.05) (Tables 3, 4, 5, 6, 7).

**Table 3:** Comparation of preoperative and postoperative WOMAC

	*n*	Preoperative WOMAC	Postoperative WOMAC	*t*	*P* value
NSP group	44	67.55 ± 10.21	15.82 ± 5.34	14.110	< 0.001
SSP group	78	68.72 ± 7.80	15.90 ± 3.68	42.530	< 0.001
*t*		− 0.411	− 0.057		
*P* value		0.683	0.955		

WOMAC, McMaster Universities osteoarthritis index

**Table 4:** Comparation of preoperative and postoperative KSS knee scores

	*n*	Preoperative KSS knee score	Postoperative KSS knee score	*t*	*P* value
NSP group	44	58.73 ± 5.87	88.09 ± 4.16	− 14.320	< 0.001
SSP group	78	57.82 ± 5.54	86.87 ± 2.68	− 27.839	< 0.001
*t*		0.474	1.172		
*P* value		0.638	0.247		

KSS, the Knee Society Score

**Table 5:** Comparation of preoperative and postoperative KSS function scores

	*n*	Preoperative KSS function score	Postoperative KSS function score	*t*	*P* value
NSP group	44	27.27 ± 13.69	83.64 ± 10.02	− 12.183	< 0.001
SSP group	78	29.62 ± 10.78	85.77 ± 5.07	− 35.669	< 0.001
*t*		− 0.600	− 0.972		
*P* value		0.552	0.336		

KSS, the Knee Society Score

**Table 6:** Comparation of preoperative and postoperative HSS scores

	*n*	Preoperative HSS score	Postoperative HSS score	*t*	*P* value
NSP group	44	46.64 ± 3.26	83.64 ± 5.78	− 20.923	< 0.001
SSP group	78	48.13 ± 3.81	86.08 ± 3.01	− 47.703	< 0.001
*t*		− 1.181	− 1.900		
*P* value		0.243	0.063		

HSS, the Hospital for Special Surgery knee score

**Table 7:** Comparation of preoperative and postoperative Lysholm knee scores

	n	Preoperative Lysholm score	Postoperative Lysholm score	*t*	*P* value
NSP group	44	59.91 ± 8.18	85.64 ± 4.80	− 8.532	< 0.001
SSP group	78	60.85 ± 5.80	85.97 ± 3.48	− 22.226	< 0.001
*t*		− 0.431	− 0.261		
*P* value		0.668	0.759		

### Postoperative change in PTS

Two independent sample t-test showed no significant difference in the Preoperative PTS between the two groups (*P* > 0.05). Paired t-test showed that, in the NSP group, PTS at the 1-day postoperative and most recent follow-up periods were significantly greater than the preoperative PTS (*t* = − 3.213, − 6.406, all *P* < 0.001), but no difference was seen in the SSP group (*P* > 0.05). Two independent sample t-tests showed that, at the 1-day postoperative and most recent follow-up periods, PTS in the NSP group were significantly greater than that in the SSP group (*t* = 1.335, *P* = 0.039; *t* = 2.888, *P* = 0.006). Compared with that in the SSP group, change in 1-day postoperative PTS and change in most recent follow-up PTS in the NSP group were significantly greater (*t* = 2.243, *P* = 0.030; *t* = 6.501, *P* < 0.001). (Table 8).

**Table 8:** Postoperative change in PTS

	Preoperative PTS (°)	1-day postoperative PTS (°)	Most recent follow-up PTS (°)	Change in 1-day postoperative PTS (°)	Change in most recent follow-up PTS (°)
NSP group	9.39 ± 1.85	10.85 ± 2.36(a)	11.74 ± 2.45(a)	0.86 ± 1.62	2.35 ± 1.21
SSP group	9.26 ± 2.34	9.07 ± 2.33(b)	9.37 ± 2.38(b)	− 0.09 ± 1.13	0.11 ± 0.95
*t*	0.166	1.335	2.888	2.243	6.501
*P* value	0.869	0.039	0.006	0.030	< 0.001

PTS, posterior tibial slope; a, difference with preoperative PTS is statistically significant; b, difference with preoperative PTS is not statistically significant; a and b are examined by paired t-tests

## Discussion

The most important finding of this study is that, compared to the use of a naturally straightened knee joint position, use of a suspended and straightened knee joint position achieved by placing a sterile cloth ball under the ipsilateral ankle raising the ankle when steel plates are fixed can effectively prevent the increase in the posterior tibial slope after OWHTO. Second, after operation, the pain symptoms were relieved and the knee joint function of the patients was markedly improved in both groups, but the two groups of patients were comparable for the degree of relief of pain and improvement of knee function without significant difference (*P* > 0.05). We suspect that, when the follow-up time is further extended, these clinical differences may appear at a statistically significant level.

According to a systematic review of open-wedge HTO studies, an increase in PTS could result in forward tibial movement relative to the femur. Studies have shown that a 1° increase in PTS is accompanied by a 1.45° increase in the loss of knee extension [15]. Pressure distribution in the knee compartments is rearranged when PTS increases by 5.5°: The peak pressure point moves 24% of the knee compartment backward, which may be an important cause of pain or surgical failure after OWHTO [16]. In addition, forward tibial movement resulting from an increase in PTS results in increased tension of the anterior cruciate ligament (ACL), which eventually aggravates the degeneration of the ACL [21], and some other studies have even confirmed that an increase in PTS is a risk factor for postoperative anterior cruciate ligament injury, which results in knee instability and accelerates knee degeneration [22]. Studies have reported that an increase in postoperative PTS increases the amount of osteotomy on the anterior tibial plateau during secondary total knee arthroplasty (TKA) [9, 23–25]. It has also been reported that an increase in PTS is accompanied by contracture of the patellar tendon, and secondary TKA is more difficult to perform [26–29]. Therefore, preventing an increased postoperative PTS is of great significance to avoid the occurrence of adverse complications after HTO.

Previous studies have made several recommendations to prevent an increase in PTS after OWHTO, including maintaining an optimal gap ratio, making a complete posterior osteotomy, and maintaining optimal hinge and steel plate positions. Lee et al. suggested that the gap ratio should maintain an anterior opening gap at approximately 67% of the posterior gap [13], and Noyes et al. found that the optimal ratio to maintain the normal sagittal tibial slope is 50% [4, 17, 18]. Dong et al. reported that autologous tricortical iliac bone grafts may be effective in achieving the optimal gap ratio [30]. Although these results are inconsistent, the researchers all aimed to keep the posterior opening gap wider than the anterior opening gap. According to Marti et al., an increase in PTS after osteotomy occurs if the posterior gap is incompletely opened or the posterior soft tissue is not fully released [3]. Joon et al. found that a lateral hinge instead of a posterolateral hinge contributes to complete opening of the posterior opening gap [16]. Ho‑Seung et al. suggested that a standard height of the hinge should be adopted to prevent an increase in PTS. The horizontal osteotomy line should be 3 cm below the medial edge of the tibial plateau toward the fibular head. A lower hinge position could result in a significant increase in postoperative PTS and an increased risk of hinge fracture [31].

Keeping the posterior gap sufficiently open is a key surgical step that should be achieved. Although distraction forceps are used to open the osteotomy gap during the operation, they achieve an appropriate postoperative force line on the coronal plane but not an optimal sagittal tibial plateau slope. A number of studies have focused on the type of osteotomy gap that should be adopted. Although knee flexors with strong strength may be resistant to posterior gap openings, the effect of knee flexors on the osteotomy gap has never been taken into serious consideration. In this study, a suspended and straightened knee joint position with a sterile cloth ball placed under the ipsilateral ankle to raise the ankle when steel plates are fixed is recommended. We suspect that such a knee joint position works by eliminating the restriction of knee flexors on the posterior gap opening and that this force is the effect of gravity on the knees. The advantage of our operation is that it is simple, easy to perform, and effective in preventing the increase in postoperative PTS. In particular, this operation is not standard or consistently performed during surgery; therefore, one of the main purposes of this study is to suggest that this procedure should be performed standardly and consistently during OWHTO.

There are several limitations to this study. First, there was a limited number of subjects. Second, other factors, such as the corrective angle, were not considered. However, the study limitations were minimized by selecting the optimal corrective angle and surgical protocols based on previously studied OWHTO outcomes. Third, other factors, such as the weight of the lower limbs, were not measured. Fortunately, we have come to a very important and practical conclusion.

## Conclusions

Open-wedge high tibial osteotomy using a suspended and straightened knee joint position by placing a sterile cloth ball under the ipsilateral ankle to raise the ankle when steel plates are fixed is suggested because it could effectively prevent an increase in postoperative PTS. With regard to clinical relevance, the use of a suspended and straightened knee joint position when fixing steel plates is critical for a satisfactory outcome of OWHTO with standardization and consistency.

## Data Availability

All data and materials comply with current standards.

## Abbreviations

PTS: Posterior tibial slope
OWHTO: Medial open-wedge high tibial osteotomy
CWHTO: Lateral closing-wedge high tibial osteotomy
BMI: Body mass index
SSP: Suspended and straightened knee joint position
NSP: Naturally straightened knee joint position
VAS: The visual analog pain scale
WOMAC: The Western Ontario and McMaster Universities osteoarthritis index
KSS: The Knee Society Score
HSS: The Hospital for Special Surgery knee score
